# Early life exposure to air pollution and psychotic-like experiences, emotional symptoms, and conduct problems in middle childhood

**DOI:** 10.1007/s00127-023-02533-w

**Published:** 2023-07-20

**Authors:** Melissa Bradley, Kimberlie Dean, Samsung Lim, Kristin R. Laurens, Felicity Harris, Stacy Tzoumakis, Kirstie O’Hare, Vaughan J. Carr, Melissa J. Green

**Affiliations:** 1 School of Clinical Medicine, Discipline of Psychiatry and Mental Health, Faculty of Medicine and Health, University of New South Wales, Sydney, NSW 2052 Australia; 2 Justice Health and Forensic Mental Health Network, Sydney, NSW Australia; 3 School of Civil and Environmental Engineering, University of New South Wales, Sydney, NSW Australia; 4 Biosecurity Program, Kirby Institute, University of New South Wales, Sydney, NSW Australia; 5 School of Psychology and Counselling, Queensland University of Technology (QUT), Brisbane, QLD Australia; 6 School of Criminology and Criminal Justice, Griffith University, Southport, QLD Australia; 7 Department of Psychiatry, Monash University, Melbourne, VIC Australia; 8 Neuroscience Research Australia, Sydney, NSW Australia

**Keywords:** Particulate matter, Nitrogen dioxide, Psychosis-proneness, Childhood mental health, Environmental risk

## Abstract

**Background:**

Air pollution has been linked to a variety of childhood mental health problems, but results are inconsistent across studies and the effect of exposure timing is unclear. We examined the associations between air pollution exposure at two time-points in early development and psychotic-like experiences (PLEs), and emotional and conduct symptoms, assessed in middle childhood (mean age 11.5 years).

**Methods:**

Participants were 19,932 children selected from the NSW Child Development Study (NSW-CDS) with available linked multi-agency data from birth, and self-reported psychotic-like experiences (PLEs) and psychopathology at age 11–12 years (middle childhood). We used binomial logistic regression to examine associations between exposure to nitrogen dioxide (NO_2_) and particulate matter less than 2.5 μm (PM_2.5_) at two time-points (birth and middle childhood) and middle childhood PLEs, and emotional and conduct symptoms, with consideration of socioeconomic status and other potential confounding factors in adjusted models.

**Results:**

In fully adjusted models, NO_2_ exposure in middle childhood was associated with concurrent PLEs (OR = 1.10, 95% CI = 1.02–1.20). Similar associations with PLEs were found for middle childhood exposure to PM_2.5_ (OR = 1.05, 95% CI = 1.01–1.09). Neither NO_2_ nor PM_2.5_ exposure was associated with emotional symptoms or conduct problems in this study.

**Conclusions:**

This study highlights the need for a better understanding of potential mechanisms of action of NO_2_ in the brain during childhood.

**Supplementary Information:**

The online version contains supplementary material available at 10.1007/s00127-023-02533-w.

## Introduction

Air pollution has been linked to an increasing array of mental health problems across the lifespan [1]. In adults, depression and (to a lesser extent) anxiety have been shown to be associated with long-term exposure to particulate matter less than 2.5 μm (PM_2.5_), and short-term exposure may be associated with completed suicide [1]. Schizophrenia (and polygenic risk for schizophrenia) has been linked to childhood exposure to nitrogen dioxide (NO_2_) [2]. In addition, perinatal exposure to airborne particulate matter, particularly PM_2.5_, has been associated with a variety of neurodevelopmental outcomes in childhood including autism spectrum and attention-deficit/hyperactivity disorders [3, 4]. While these neurodevelopmental disorders are diagnosed in childhood, mental disorders such as schizophrenia are rarely expressed in childhood, making the trajectory of their association with childhood air pollution exposure difficult to trace [5]. However, adult mental disorder is commonly preceded by childhood psychopathology [6], and childhood emotional and behavioural symptoms and psychotic-like experiences can be measured by self-report [7] for examination in relation to air pollution exposure.

Childhood Psychotic-like Experiences (PLEs) are relatively common, but children with more frequent or severe PLEs have a higher risk of developing schizophrenia in adulthood compared to children with more transient or no experience of PLEs [8]. Only one previous study has examined the association between self-reported PLEs in childhood (using a Prodromal Questionnaire-Brief Child Version at age 9–10 years) and prior exposure to NO_2_ and PM_2.5_, reporting no association in models adjusted for a number of variables, including parental PLEs, family mental health history, and financial adversity [9]. There has also been one study of these associations in relation to PLEs measured in adolescence: in a cohort of twins, exposure to NO_2_ and PM_2.5_ at age 18 years were each associated with psychotic experiences at the same age [10]_,_ after adjusting for a range of covariates including urbanicity, socioeconomic status, substance misuse, and maternal psychosis. In addition to the above studies examining childhood and adolescent PLEs, prenatal exposure to polycyclic aromatic hydrocarbons (PAHs), produced by combustion of carbon-based compounds, was associated with early childhood adversity and a more general construct of *thought problems* (which included some PLEs) on Achenbach’s Child Behaviour Checklist (CBCL) in middle childhood [11].

More studies have examined associations between air pollution exposure and emotional symptoms in childhood, although results have been mixed. For example, only two studies reported a positive association between air pollution exposure in the perinatal period and depressive/anxiety symptoms. Perinatal PAH exposure was associated with parent-rated symptoms at age 6–7 years [12] and perinatal (but not concurrent) traffic exposure was associated with parent, but not child-rated, symptoms age 12 years [13]. Five other studies have reported no association between air pollution exposure in the perinatal period and childhood depressive/anxiety symptoms [9, 14–17]. The effects of air pollution exposure in later childhood also remain unclear: exposure to NO_2_ and PM_2.5_ at age 12 years was not associated with concurrent depressive/anxiety symptoms but was associated with these symptoms at age 18 years [18].

Similarly, conduct symptoms have been associated with air pollution in some studies but not others. Perinatal exposure to PM_2.5_ and PAH has been associated with conduct symptoms in children ranging from 6 to 9 years [19]. For example, NO_2_ exposure at 9 months showed a small association with parent-rated conduct problems at age 3 years [20]. However, there was a lack of association between perinatal air pollution and behavioural problems in a Japanese cohort at age 8 [21]. Studies of later air pollution exposure and conduct symptoms have produced more mixed results. There was a strong association between exposure to PM_2.5_ (but not NO_2_) in the preceding 1–3 years and delinquent behaviour in a study of children and adolescents [22], and there was an improved trajectory of self-reported conduct problems on the SDQ in middle school age children exposed to lower levels of NO_2_ (as well as PM_2.5_) in the preceding years [23]. However, a study of 9–10 years old found no association between exposure to PM_2.5_ and conduct problems around the same age [9]. Further, in a twin cohort, there was no association between age 12 exposure to PM_2.5_ or NO_2_ and conduct symptoms at age 12 or 18 years [10],

Given childhood PLEs have been sparsely studied in relation to air pollution despite known association with later psychotic symptoms and schizophrenia, we examined the associations between multiple types of air pollution and child reported PLEs in middle childhood (average age 11.5 years) using a large, population-based study of children in New South Wales (NSW) Australia. We also examined associations between air pollution and other mental health domains (emotional symptoms and conduct problems) to determine whether any associations with PLEs were limited to this construct, or more generally associated with childhood psychopathology. Given the heterogeneous results of previous studies which measured air pollution at ages varying from birth to 18 years, we chose two developmentally important time-points of exposure: birth and middle childhood (age 11.5 years). We considered several covariates used in the previous studies, including socioeconomic status, parental history of mental illness, and urbanicity [10]. Aboriginal or Torres Strait Islander status was also considered, because Indigenous people are overrepresented in community cohort studies of mental disorders [24]. We hypothesised that NO_2_ would be more strongly associated with PLEs than emotional or conduct symptoms, given the consistent literature linking NO_2_ with psychotic symptoms in a range of contexts. Further, we hypothesised that PM_2.5_ would be most associated with conduct symptoms. We made no specific hypotheses about expected effects of the different exposure periods (birth or middle childhood) given mixed evidence of associations between air pollutants and childhood mental health symptoms in various developmental periods of exposure.

## Methods

### Sample and procedure

**Fig. 1:**
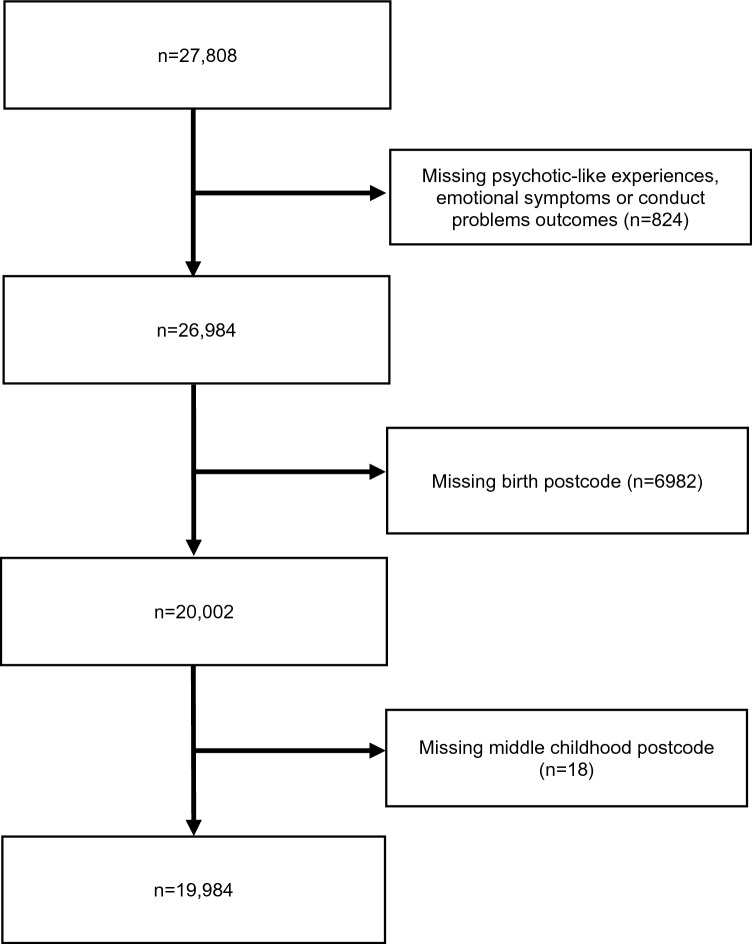
Sample selection

Child participants in this study were drawn from the New South Wales Child Development Study (NSW-CDS), a multi-agency longitudinal record-linkage study of 99.7% of children who started school in New South Wales (NSW) in 2009 [25]. The second wave of linkage for this cohort [26] included data for 27,808 children who took part in the Middle Childhood Survey (MCS) in 2015, a self-report mental health and emotional well-being survey [27]. Schools were invited to take part in the MCS via a letter to school leaders and 829 (35% of eligible) schools in NSW agreed to administer the MCS in school time; 85.9% of children at participating schools took part in the MCS, with non-participation due to care-giver or child opt-out, absenteeism on day of the survey, or data-platform failure [27]. At the time of the survey, the children had a mean age of 11.5 years and were in their final year of primary (elementary) school. From this sample of children who completed the MCS, the present study included 19,984 children who answered all questions relating to PLEs, emotional symptoms, and conduct problems on the MCS, and for whom residential area data were available at two time-points (i.e., at birth and MCS administration; Fig. 1). There were no missing covariate data for the selected sample.

Data linkages were conducted by the Centre for Health Record Linkage (CHeReL: www.cherel.org.au/) using probabilistic methods, with a false positive linkage rate < 0.5%. Researchers had access only to de-identified data and were unable to report cell sizes < 15 to protect the privacy of participants. Ethical approvals were obtained from the NSW Population and Health Services Research Ethics Committee (HREC/11/CIPHS/14) for the record linkage, and from the University of New South Wales Human Research Ethics Committee for the administration of the MCS (HC11409).

### Outcome measures

#### Psychotic-like experiences

Children answered nine questions within the MCS relating to PLEs, from the Psychotic-Like Experiences Questionnaire for Children (PLEQ-C) [28]; there were two minor changes of wording from the original scale to improve comprehensibility for the Australian population. Of the nine questions, five questions were adapted from the Diagnostic Interview Schedule for Children [29], and an additional four items assessed a broader range of PLEs. Available responses were “Not True”, “Somewhat True”, or “Certainly True”, following the format of responses on the Strengths and Difficulties Questionnaire (SDQ). These responses were rated on a 3-point scale (0 = not true, 1 = somewhat true, and 2 = certainly true). The PLE sub-scale had excellent ordinal alpha reliability in the current sample (*a* = 0.90). To mirror existing normative categories for emotional problems and conduct symptoms, we summed the total score on PLEQ-C (range 0–18) and categorised scores in the top 20% of the population as high and the bottom 80% as normal; this threshold was chosen to match both known rates of PLEs at clinical interview in this age group, as well as being comparable to the size of established normative categories for emotional symptoms and conduct problems. Previous research has determined a median rate of PLEs at interview for this age group of 17% for 9–12 years old [30]. In addition, the use of cut-off scores on PLE screening questionnaires has been found to have good sensitivity and specificity for predicting PLEs validated on interview [31]. Since the screening instrument was effectively a Likert scale (being the sum of Likert scales), sensitivity analysis using a full-scale rather than cut-off outcome was proposed should an effect be found. For sensitivity analysis, the total PLE score (0–18) was utilised as an outcome variable, making no assumptions about relative distance between scores.

#### Emotional symptoms and conduct problems

Emotional symptoms and conduct problems were assessed within the MCS at a single time-point (2015) using the Emotional Symptoms and Conduct Problems sub-scales from the self-reported SDQ, respectively; responses to the five questions from each sub-scale were summed (“Not True”, “Somewhat True”, and “Certainly True” corresponding to 0, 1, or 2). Ordinal alphas for the Emotional Symptoms and Conduct Problem sub-scales with the current sample were 0.79 and 0.77, respectively. The total scores for each sub-scale were converted to three levels of outcome, according to the original normative categories developed by Goodman [32] based on a UK population sample. For the Emotional Symptoms sub-scale, scoring below 5 is taken to represent the “normal” band, 6 is deemed “borderline”, and 7–10 is taken to represent the “abnormal” band. For the Conduct Problems sub-scale, the “normal” band is 0–3, “borderline” band is 4, and “abnormal” band 5–10. For both indices, we converted these three categories to a dichotomous outcome variable by merging the “borderline” and “abnormal” bands to form a “high” category, for comparison with the ‘normal’ band, for each sub-scale.

### Exposure measures

#### Air pollution

The place of residence of each child was determined at two time-points—at birth and middle childhood—using postcode information available at birth from the NSW Ministry of Health’s Perinatal Data Collection (PDC) and in middle childhood from the MCS (mean age 11.5 years). These location data were then used to estimate PM_2.5_ and NO_2_ exposure for each child from publicly available data. The median residential postcode area for the children at the time of the MCS was 32.5 km^2^.

Ground-level pollution across NSW was estimated from two types of publicly available data: a network of ground monitoring stations and data sets derived from satellite measurements. We used data fusion, a well-established statistical technique [33] widely used to integrate environmental data, to combine these data sources and derive ground-level air pollution concentrations according to area-based information for each child living in NSW. There were two methods required to estimate air pollution concentrations for regions inside the Greater Sydney region, versus regions outside Greater Sydney, because the ground monitoring stations are densely populated in the Greater Sydney region but sparse in the regions outside Greater Sydney.

For the Greater Sydney region defined as per Greater Capital City Statistical Area (Australian Bureau of Statistics, 2017), air pollution concentration maps were constructed using ground data alone and Inverse Distance Weighted (IDW) interpolation. Ground data were available from the NSW Air Quality Monitoring Network which is maintained by the Climate and Atmospheric Science Branch, Department of Planning, industry and Environment (DPIE) of the NSW Government [34]. We used adaptive neighbourhood search techniques to find a minimum of ten and a maximum of fifteen monitoring stations within the neighbourhood to apply IDW interpolation and construct a pollution concentration map across Greater Sydney. Annual average ground-level concentrations of PM_2.5_ were reported in micrograms per metre cubed (µgm/m^3^) and annual average NO_2_ concentration were reported in units of parts per hundred million (pphm). These were then averaged across postcode areas according to Australian Statistical Geography Standard (ASGS) boundaries [35].

For areas outside of Greater Sydney, a data fusion technique was used to estimate ground pollution using satellite data obtained from the OMNO2 data set [36]. This data set provides a map of daily average NO_2_ ground-level concentration across the globe at 0.25 × 0.25 degree resolution, constructed by combining a range of satellite data with local meteorological parameters. Satellite data for PM_2.5_ ground-level concentration were obtained from the Modern-Era retrospective analysis for Research and Applications version 2 (MERRA-2) data set [37]. The PM_2.5_ satellite data were obtained as monthly averages, at 0.5 × 0.625 degree resolution, constructed from a range of satellite data together with local meteorological data. These satellite data sets provided estimates of ground-level concentrations, which were then converted to the same units as the ground monitoring data (µgm/m^3^ for PM_2.5_ and pphm for NO_2_) and integrated over postcodes according to ASGS boundaries. Annual averages were then obtained. To find an appropriate conversion factor which would allow fusion of satellite data with ground monitoring station data, we calculated a polynomial regression coefficient by comparing satellite data over Greater Sydney with the data obtained by IDW from Ground Monitoring Station data also covering Greater Sydney. Using regression techniques to minimise Root-Mean-Square Error (RMSE), we found the optimal polynomial regression coefficient, and used this to transform the satellite data across the areas of NSW outside of Greater Sydney. Normalised RMSE using this technique ranged from 6 to 18%, and were greater for PM_2.5_ than NO_2._ This was expected as satellite data sets of PM_2.5_ ground concentrations are thought to be more error-prone than satellite data sets of NO_2_ [38].

### Covariates

#### Urbanicity

Urbanicity was estimated from residential postcode in the MCS (2015) in conjunction with Australian Census Data collected in 2016. Urbanicity refers to the degree of urbanisation [39] which can be conveniently estimated by population density. We used the 2016 Census Time Series Profile that provides population changes over three time-points: 2006, 2011, and 2016 [40]. The unit of urbanicity in this study is reported as population per squared kilometres. Urbanicity scores initially intended to be linear but were divided into quartiles after initial data visualisation of “U-shaped” scatter plots on scatter plots (plotted as urbanicity versus outcome measures).

#### Socio-economic status

Socio-economic status, based on residential postcode in the MCS, was dichotomised as the lower three quintiles (most disadvantaged) versus upper two quintiles (least disadvantaged) on the Index for Relative Socio-economic Disadvantage according to the Australian 2016 Socio-Economic Indexes for Areas (SEIFA) [40].

#### Parental history of mental disorder

Records of parental mental disorder diagnoses were obtained from the NSW Ministry of Health’s Admitted Patient Data Collection (APDC), Mental Health Ambulatory Data Collection (MH-AMB), and Emergency Department Data Collection (EDDC). The APDC details admitted patient diagnoses, procedures, and services provided by NSW public and private hospitals, and day procedure centres from July 2001 to June 2016. The MH-AMB includes records of ambulatory public mental health services, including outpatient services, from January 2000 to December 2016. The EDDC includes information on emergency department presentations from the majority of NSW public hospitals between January 2005 and June 2016. We considered that there was a positive history of mental disorder if either parent had a recorded mental disorder in any of the data sets, according to F-codes of the International Statistical Classification of Diseases and Related Health Problems (World Health Organization, 1992) Tenth Revision, Australian Modification (ICD-10-AM).

#### Aboriginal or Torres Strait Islander status

Aboriginal or Torres Strait Islander status was determined for each child if recorded in any of the data collections for the child, their mother, or father.

### Statistical analysis

All statistical analyses were conducted using R version 4.1.0 (2021-05-18) [41] in R Studio [42]. A series of unadjusted and adjusted binomial logistic regressions were used to estimate associations between each of the air pollutants and the outcome variables. Adjusted models included all sociodemographic covariates. For each outcome variable (PLEs, emotional symptoms, and conduct problems), we calculated the odds ratios and 95% confidence intervals for a one interquartile range increase in average air pollutant concentration. ORs between 1.00 and 1.49 (1.00–0.67) were considered small, 1.50 and 2.49 (0.66–0.40) as medium, and 2.50 (< 0.40) or over as large [43]. Given correlation between pollutants, as well as correlation across time of individual pollutants, additional sensitivity analyses were carried out to explore associations with: exposure to each air pollutant measured at both time-points in the same model/s (Supplementary Table 1); exposure to both pollutants in middle childhood (Supplementary Table 2), and an alternate PLE outcome (total PLE score considered as ordinal value; Supplementary Table 3). Details of methodology and results for all sensitivity analyses are provided in Supplementary Materials.

## Results

### Sociodemographic characteristics of sample

Table 1 summarises the sociodemographic characteristics and environmental exposure level of the entire sample and by category of PLEs, emotional symptoms and conduct problems.

**Table 1:** Sociodemographic characteristics

	All (*N* = 19,984)	Psychotic-like experiences (PLEs)		Emotional symptoms		Conduct problems	
		Low PLEs (*n* = 16,036)	High PLEs (*n* = 3948)	Low emotional symptoms (*n* = 16903)	High emotional symptoms (*n* = 3081)	Low conduct problems (*n* = 16,649)	High conduct problems (*n* = 3335)
Variable	*N* (%)	*N* (%)	*N* (%)	*N* (%)	*N* (%)	*N* (%)	*N* (%)
**Gender**							
Female	9988 (50)	7945 (80)	2043 (20)	8083 (81)	1905 (19)	8720 (87)	1268 (13)
Male	9996 (50)	8091 (81)	1905 (19)	8820 (88)	1176 (12)	7929 (79)	2067 (21)
**Socio-economic status**							
Low/medium	11,865 (59)	10,552 (81)	2535 (19)	9901 (83)	1964 (17)	9609 (81)	2256 (19)
High	8119 (41)	5484 (80)	1413 (20)	7002 (86)	1117 (14)	7040 (87)	1079 (13)
**Aboriginal or Torres Strait Islander**							
Yes	1324 (6)	1026 (77)	298 (23)	1063 (80)	261 (20)	952 (72)	372 (28)
No	18,660 (93)	15,010 (80)	3650 (20)	15,840 (85)	2820 (15)	15,697 (84)	2963 (16)
**Parental mental disorder**							
Yes	4626 (23)	459 (78)	128 (22)	3724 (81)	902 (19)	3614 (78)	1012 (22)
No	15,358 (77)	15,577 (80)	3820 (20)	13,179 (86)	2179 (14)	13,035 (85)	2323 (15)
**Urbanicity**							
Lowest 25%	4996 (25)	4053(81)	943 (19)	4250 (85)	746 (15)	4051 (81)	945 (19)
> 25% and ≤ 50%	4996 (25)	3958 (79)	1038 (21)	4219 (84)	777 (16)	4133 (83)	863 (17)
> 50% and ≤ 74%	4996 (25)	3958 (79)	1038 (21)	4169 (83)	827 (17)	4183 (84)	813 (16)
Highest 25%	4996 (25)	4067 (81)	929(19)	4265 (85)	731 (15)	4282 (86)	714 (14)

Air pollution	Mean (SDa)	Mean (SD)	Mean (SD)	Mean (SD)	Mean (SD)	Mean (SD)	Mean (SD)
**NO**_2_ in pphm^b^							
Birth	0.90 (0.43)	0.90 (0.42)	0.90 (0.42)	0.90 (0.43)	0.90 (0.43)	0.91 (0.43)	0.86 (0.44)
Mid-childhood	0.78 (0.34)	0.79 (0.33)	0.79 (0.33)	0.78 (0.34)	0.78 (0.34)	0.79 (0.34)	0.75 (0.35)
**PM**_2.5_ in μg/m^3c^							
Birth	7.19 (1.29)	7.21 (1.24)	7.23 (1.27)	7.20 (1.28)	7.19 (1.29)	7.21 (1.27)	7.13 (1.32)
Mid-childhood	7.01 (0.99)	7.05 (0.96)	7.04 (0.96)	7.01 (0.98)	7.03 (1.00)	7.03 (0.98)	6.96 (1.04)

^a^Standard deviation

^b^Nitrogen dioxide in parts per hundred million

^c^Particulate matter less than 2.5 μm in micrograms per metre cubed

Mean estimated NO_2_ exposure for the children, averaged over the years of birth and middle childhood in parts per hundred million were 0.90 (IQR = 0.43) and 0.78 (IQR = 0.34), respectively. These exceed WHO recommended annual average concentrations of 10 µg per metre cubed, or approximately 0.5 pphm [44]. Mean estimated PM_2.5_ concentrations averaged over each of the years of birth and middle childhood in micrograms per metre cubed (μg/m^3^) were 7.20 (IQR = 1.28) and 7.02 (IQR = 0.99), respectively. Again, these exceeded WHO recommended annual mean concentrations of 5 μg/m^3^ [44]. There was significant correlation between NO_2_ and PM_2.5_ at both exposure time-points. The correlation coefficient for NO_2_ exposure at birth and PM_2.5_ exposure at birth was 0.77; in middle childhood, this coefficient was 0.75. Just over half (10,144; 51%) of children had the same residential suburb recorded at birth and MCS administration.

### Air pollution and PLEs

NO_2_ exposure was not associated with PLEs in unadjusted models (birth exposure OR = 1.02, CI 0.98–1.06; middle childhood exposure OR = 1.03, CI 0.99–1.08). However, after adjusting for covariates, there were small but statistically significant associations between exposure to nitrogen dioxide in middle childhood only and PLEs (Table 2). Exposure to PM_2.5_ in middle childhood only was associated with PLEs in unadjusted (birth exposure OR = 1.01, CI 0.99–1.04; middle childhood exposure OR = 1.04, CI 1.01–1.06) and adjusted models, again with small odds ratios (Table 2).

**Table 2:** Associations between air pollutants and psychotic-like experiences

Variable	Odds ratio (95% CI^a^) for birth model		Odds ratio (95% CI) for middle childhood model	
	Adjusted^b^ NO_2_c	Adjusted^b^ PM_2.5_d	Adjusted^b^ NO_2_	Adjusted^b^ PM_2.5_
**NO**_2_	1.04 (0.98–1.11)	–	1.10 (1.02–1.20)	–
**PM**_2.5_	–	1.02 (0.98–1.05)	–	1.05 (1.01–1.09)
**Socio-economic status**				
High	REF	REF	REF	REF
Low/medium	1.09 (1.01–1.17)	1.08 (1.01–1.17)	1.09 (1.01–1.18)	1.10 (1.04–1.17)
**Aboriginal or Torres Strait Islander**				
No	REF	REF	REF	REF
Yes	1.19 (1.03–1.36)	1.18 (1.03–1.36)	1.19 (1.04–1.37)	1.19 (1.07–1.34)
**Parental mental disorder**				
No	REF	REF	REF	REF
Yes	1.10 (0.89–1.33)	1.10 (0.89–1.33)	1.09 (0.89–1.33)	1.05 (0.89–1.25)
**Urbanicity**				
Lowest 25%	REF	REF	REF	REF
> 25% & ≤ 50%	1.12 (1.00–1.25)	1.14 (1.02–1.247)	1.08 (0.96–1.21)	1.08 (0.96–1.21)
> 50% & ≤ 74%	1.10 (0.96–1.26)	1.13 (1.00–1.27)	1.03 (0.90–1.19)	1.06 (0.94–1.20)
Highest 25%	0.95 (0.81–1.10)	0.99 (0.87–1.12)	0.87 (0.74–1.03)	0.93 (0.82–1.05)

^a^Confidence interval

^b^Adjusted for socioeconomic status, Aboriginal or Torres Strait Islander status, parental history of mental disorder, and urbanicity

^c^Nitrogen dioxide

^d^Particulate matter less than 2.5 μm

When exposures to NO_2_ at both birth and middle childhood were included in the same regression model, the odds ratios remained similar (birth exposure OR = 0.90, CI 0.80–1.03; middle childhood exposure OR = 1.23, CI 1.05–1.44; Supplementary Table 1). However, when both birth and middle childhood exposures to PM_2.5_ were included in the same model, only birth exposure (OR = 1.07, CI 1.02–1.13), but not middle childhood exposure (OR = 0.97, CI 0.93–1.02) was significantly associated with PLEs (Supplementary Table 1). Two pollutant models were also examined in association with PLEs (Supplementary Table 2), but there were no statistically significant associations for either pollutant when both were included in the same models, for either the birth or middle childhood exposure period. A final sensitivity analysis was conducted to explore the use of total PLE scores on 9 PLE items, considered as an ordinal outcome measure (Supplementary Table 3). Odds ratios were comparable to those found for the binary PLE outcome measure: birth exposure to pollutants was associated with PLEs for both NO_2_ (OR = 1.08; CI 1.03–1.12) and PM_2.5_ (OR = 1.06; CI 1.03–1.09). Exposure to NO_2_ in middle childhood was also associated with PLEs in fully adjusted ordinal models (OR = 1.12; CI 1.06–1.18), as was exposure to PM_2.5_ (OR = 1.08; CI 1.06–1.11).

### Air pollution and emotional symptoms

There were no statistically significant associations between exposure to NO_2_ at any time-point and emotional symptoms in unadjusted (birth exposure OR = 0.98, CI 0.90–1.07; middle childhood exposure OR = 1.01, CI 0.96–1.06) or adjusted models (Table 3). Similarly, there was no association with PM_2.5_ in unadjusted (birth exposure OR = 0.99, CI 0.97–1.03; middle childhood exposure OR = 1.01, CI 0.98–1.04) or adjusted models (Table 3).

**Table 3:** Associations between air pollutants and emotional symptoms

Variable	Odds ratio (95% CI^a^) for birth model		Odds ratio (95% CI) for middle childhood model	
	Adjusted^b^ NO_2_^c^	Adjusted^b^ PM_2.5_^d^	Adjusted^b^ NO_2_	Adjusted^b^ PM_2.5_
**NO**_2_	0.97 (0.84–1.12)	–	1.04 (0.95–1.14)	–
**PM**_2.5_	–	0.99 (0.95–1.03)	–	1.02 (0.98–1.06)
**Socio-economic status**				
High	REF	REF	REF	REF
Low/medium	1.24 (1.14–1.35)	1.24 (1.15–1.35)	1.25 (1.15–1.36)	1.25 (1.15–1.35)
**Aboriginal or Torres Strait Islander**				
No	REF	REF	REF	REF
Yes	1.32 (1.14–1.52)	1.32 (1.14–1.52)	1.33 (1.15–1.53)	1.33 (1.14–1.53)
**Parental mental disorder**				
No	REF	REF	REF	REF
Yes	1.28 (1.03–1.57)	1.28 (1.03–1.57)	1.28 (1.03–1.57)	1.28 (1.03–1.57)
**Urbanicity**				
Lowest 25%	REF	REF	REF	REF
> 25% & ≤ 50%	1.13 (0.99–1.28)	1.12 (1.00–1.27)	1.08 (0.95–1.23)	1.08 (0.95–1.23)
> 50% & ≤ 74%	1.24 (1.07–1.45)	1.24 (1.08–1.41)	1.16 (0.99–1.35)	1.17 (1.02–1.34)
Highest 25%	1.10(0.93–1.30)	1.08 (0.95–1.24)	1.00 (0.83–1.19)	1.02 (0.89–1.18)

^a^Confidence interval

^b^Adjusted for socioeconomic status, Aboriginal or Torres Strait Islander status, parental history of mental disorder, and urbanicity

^c^Nitrogen dioxide

^d^Particulate matter less than 2.5 μm

### Air pollution and conduct symptoms

In unadjusted models, NO_2_ exposure at all time-points was significantly associated with reduced conduct symptoms in middle childhood (birth exposure OR = 0.88, CI 0.85–0.92; middle childhood exposure OR = 0.88, CI 0.83–0.93). There was a similar pattern for PM_2.5_ exposure in unadjusted models (birth exposure OR = 0.95, CI 0.93–0.98; middle childhood exposure OR = 0.95, CI 0.93–0.98). However, after adjusting for covariates, there was no statistically significant association (Table 4).

**Table 4:** Association between air pollutants and conduct problems

Variable	Odds ratio (95% CI^a^) for birth model		Odds ratio (95% CI) for middle childhood model	
	Adjusted^b^ NO_2_^c^	Adjusted^b^ PM_2.5_^d^	Adjusted^b^ NO_2_	Adjusted^b^ PM_2.5_
**NO**_2_	1.00(0.93–1.07)	–	1.08 (0.99–1.18)	–
**PM**_2.5_	–	1.03 (0.99–1.07)	-	1.04 (1.00–1.08)
**Socio-economic status**				
High	REF	REF	REF	REF
Low/medium	1.45 (1.34–1.57)	1.46 (1.35–1.57)	1.45 (1.33–1.58)	1.45 (1.33–1.57)
**Aboriginal or Torres Strait Islander**				
No	REF	REF	REF	REF
Yes	1.82 (1.59–2.07)	1.83 (1.61–2.08)	1.84 (1.64–2.10)	1.84 (1.62–2.09)
**Parental mental disorder**				
No	REF	REF	REF	REF
Yes	1.26 (1.03–1.54)	1.26 (1.03–1.54)	1.27 (1.03–1.55)	1.27 (1.03–1.55)
**Urbanicity**				
Lowest 25%	REF	REF	REF	REF
> 25% & ≤ 50%	1.03 (0.91–1.16)	0.99 (0.89–1.11)	0.94 (0.83–1.05)	0.93 (0.82–1.04)
> 50% & ≤ 74%	0.97 (0.84–1.12)	0.92 (0.81–1.04)	0.90 (0.77–1.04)	0.91 (0.79–1.03)
Highest 25%	0.85 (0.72–1.01)	0.80 (0.71–0.92)	0.76 (0.64–0.91)	0.79 (0.69–0.91)

^a^Confidence interval

^b^Adjusted for socioeconomic status, Aboriginal or Torres Strait Islander status, parental history of mental disorder, and urbanicity

^c^Nitrogen dioxide

^d^Particulate matter less than 2.5 μm

## Discussion

This population cohort study of the association between air pollution exposure at two time-points (birth and middle childhood) and self-reported PLEs, emotional and conduct symptoms at a single time-point (mean age 11.5 years), revealed positive associations between exposure to NO_2,_ and to a lesser extent PM_2.5_, in middle childhood and concurrent PLEs. This is a novel finding and, if replicated, raises the possibility that exposure to NO_2_ in the atmosphere could be involved in the development of psychotic-like experiences. It must be noted that effect sizes were small and as such, the clinical implications are uncertain given the unclear relationships between PLEs and later psychotic or other mental disorders. The lack of association between air pollution and middle childhood emotional symptoms and conduct problems in adjusted models suggests some specificity of this association. Further research is needed to identify if certain groups of children are more vulnerable to increased PLEs when exposed to NO_2_, and how this relates to later schizophrenia risk.

The small but significant association between exposure to NO_2_ and PLEs in middle childhood is consistent with (but smaller than) previously reported associations between PLEs in late adolescence and concurrent exposure to NO_2_ [10], but inconsistent with a study of PLEs in 9–10 years old, where no association was found with exposure to PM_2.5_ or NO_2_ [9]. However, in the latter study, NO_2_ exposure was inferred from residential location at the same time as the PLE assessment (2016–2018) and then estimated using satellite data averaged over 3 years from 2010 to 2012; this may have introduced some measurement error, particularly if children had moved during those years. Given the small effect size in the current study, it seems plausible such a measurement error could obscure a small association. In addition, the current study suggests proximal exposure to NO_2_ may be more important than more distal exposure (such as perinatal exposure), so that a study based on exposure many years prior may not detect a result. The similarity of the screening instruments, and the validity of using such instruments to assess PLEs suggest the choice of instrument is unlikely to significantly affect outcome [31]; the robust current result, persisting with ordinal regression, suggests that the choice of outcome variable does not explain the inconsistent results across studies.

This study failed to replicate the previous findings of an association between perinatal NO_2_ exposure and conduct symptoms [20] (as well as more general externalising symptoms [17]). Outcomes in these studies were measured at a much earlier age (preschool rather than middle childhood) and earlier onset symptoms may be more associated with air pollution. Our results are also inconsistent with a study demonstrating that NO_2_ exposure in preceding years alters the trajectory of conduct problems in middle childhood [17]. However, the current study did not examine the trajectory of conduct symptoms and it may be that air pollution influences trajectories of higher risk children, rather than cross-sectional conduct problem scores in the general population.

The lack of association between air pollution and emotional symptoms accords with the majority of studies in the area [14–17]. One study which did find an association with childhood depressive symptoms was focused on a specific component of air pollution (PAH) that was not separately considered in this study [12]. It may be that only certain components of PM_2.5_ are associated with emotional symptoms and local differences in PM_2.5_ composition may alter associations. The composition of PM_2.5_ in Sydney in the summer of 2011 was around one-third sea salt and one-third organic matter with the remainder a mixture of soil, inorganic aerosols, and carbon; in autumn 2012, the mixture was almost two-thirds organic matter and only 5% sea salt [45]. More detailed modelling of PM_2.5_ components could help understanding of potential risks.

Since this study revealed associations between NO_2_ exposure and some categories of childhood symptoms but not others, this raises questions about the possible mechanisms of action of NO_2._ Proposed mechanisms, to date, have been based on post-mortem studies [46] and animal models [47] and centre on oxidative stress and cytokine release causing cell damage, after uptake through the olfactory nerve, or translocation from lungs and other organs [48, 49]. Inflammation of the olfactory bulb has been linked to high air pollution exposure, though it seems likely other central nervous system sites are also affected [50]. Studies on mechanisms have generally focused on PM_2.5,_ or air pollution as a whole, rather than NO_2_. Further studies into the mechanism of action of NO_2_ in children in conjunction with the existing research into general pollution and PM_2.5_ in children [46, 51, 52] could help address this question.

Strengths of this study include the large number of participants, the prospective, population-based design, and the access to linked data enabling adjustment for a number of key contributing factors. In the sampling, migrant populations were likely to be under-represented due to exclusion of those without a birth postcode in NSW. The other main methodological limitation of the current study relates to the measurement of pollution exposure. Exposure measures were assessed across large areas, which may have introduced measurement error. Several previous studies have used specific addresses and fine modelling to assess air pollution [18] and this is preferable to area-based estimates. The effect sizes in the current study are small and it is difficult to ascertain if they represent a small underlying association or are attenuated due to misclassification error. Finally, potential confounders may have been either unmeasured or inadequately measured, for example exposure to cigarette smoke, which is a major source of indoor NO_2_ and more subtle sociodemographic factors not captured by the reported socioeconomic level in the current study (for example, neighbourhood social cohesion, parental education level, and domestic violence).

In conclusion, the findings of this study contribute to the increasing evidence for association between NO_2_ exposure and risk for psychosis, and are the first to our knowledge to demonstrate this association in relation to childhood PLEs. The association between NO_2_ exposure and PLEs but not emotional symptoms or conduct problems raises questions about the mechanism of action of NO_2_. Furthermore, the results of this study may support the need for environmental regulation of air pollution to improve childhood mental health [4].

### Supplementary Information

Below is the link to the electronic supplementary material.

Supplementary file1 (DOCX 6893 KB)

## Data Availability

The linked data used in this study cannot be shared with third parties, owing to restrictions placed on access to Australian government administrative data to protect the privacy of participants.
