# Supplementary material for: Early life exposure to air pollution and psychotic-like experiences, emotional symptoms, and conduct problems in middle childhood
Source: Soc Psychiatry Psychiatr Epidemiol. 2023 Jul 20;59(1):87–98. doi: 10.1007/s00127-023-02533-w (PMC10799785; doi:10.1007/s00127-023-02533-w)
Supplement: Supplementary file 1 — Supplementary file1 (DOCX 6893 KB) [file 127_2023_2533_MOESM1_ESM.docx]

**Early life exposure to air pollution and psychotic-like experiences, emotional symptoms, and conduct problems in middle childhood**

**SUPPLEMENTARY MATERIALS**

Melissa Bradley^1^, Kimberlie Dean^1,2^, Samsung Lim^3,4^, Kristin Laurens^5^, Felicity Harris^1^, Stacy Tzoumakis^6^, Kirstie O’Hare^1^, Vaughan Carr^1,7,8^, Melissa Green^1,8^.

^1^Discipline of Psychiatry and Mental Health, School of Clinical Medicine, University of New South Wales, Sydney, AUSTRALIA

^2^Justice Health and Forensic Mental Health Network, Sydney, New South Wales, AUSTRALIA

^3^School of Civil and Environmental Engineering, University of New South Wales, Sydney, AUSTRALIA

^4^Biosecurity Program, Kirby Institute, University of New South Wales, Sydney, AUSTRALIA

^5^ Queensland University of Technology (QUT), School of Psychology and Counselling, Brisbane, AUSTRALIA

^6^School of Criminology and Criminal Justice, Griffith University, Southport, AUSTRALIA

^7^Department of Psychiatry, Monash University, Melbourne, AUSTRALIA

^8^Neuroscience Research Australia, Sydney, AUSTRALIA

**Table of Contents:**

Supplementary Figure 1 Map of nitrogen dioxide air pollution by postcode across Greater Sydney in 2015………………………………..………..……...p. 2

Supplementary Figure 2 Map of particulate matter (less than 2.5 microns) air pollution by postcode across Greater Sydney in 2015…………..p. 3

Supplementary Figure 3 Map of nitrogen dioxide air pollution by postcode across New South Wales in 2015……………………………..………..……..p. 4

Supplementary Figure 4 Map of particulate matter (less than 2.5 microns) air pollution by postcode across New South Wales in 2015……....p. 5

Sensitivity analysis: Air pollution exposure across birth and middle childhood in association with psychotic-like experiences…….…………….….p. 6

Sensitivity analysis: Middle childhood exposure to NO_2_ and PM_2.5_ pollution in association with psychotic-like experiences …….………..……..….p. 7

Sensitivity analysis: Use of total scores on the psychotic-like experiences (PLEs) index as an ordinal outcome measure…….……………..………….p. 8

**Nitrogen dioxide (NO_2_) air pollution by postcode across Greater Sydney in 2015**

NO_2_ levels were averaged for each postcode area in Greater Sydney using inverse distance weighted interpolation of ground monitoring station data (see Methods). Annual averages were derived for year of birth, as well as 2015 (year of Middle Childhood Survey). Map of NO_2_ levels for 2015 is shown below.

Supplementary Figure 1. Map of nitrogen dioxide air pollution by postcode across Greater Sydney in 2015


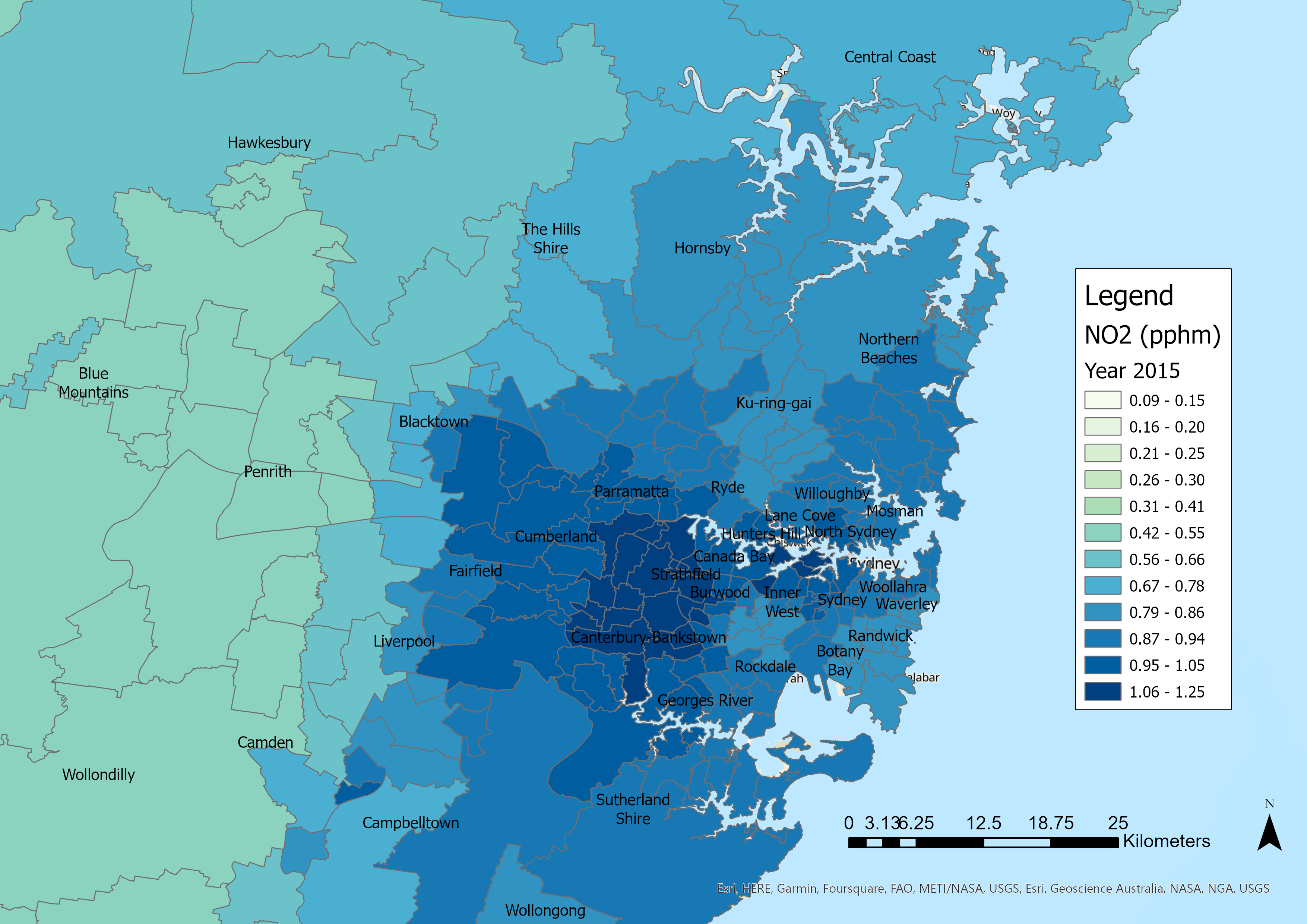


**Particulate matter less than 2.5 microns (PM_2.5_) air pollution by postcode across Greater Sydney in 2015**

PM_2.5_ levels were averaged for each postcode in Greater Sydney using inverse distance weighted interpolation of ground monitoring station data (see Methods). Annual averages were derived for year of birth, as well as 2015 (year of Middle Childhood Survey). Map of NO_2_ levels for 2015 is shown below.

Supplementary Figure 2. Map of particulate matter (less than 2.5 microns) air pollution by postcode across Greater Sydney in 2015


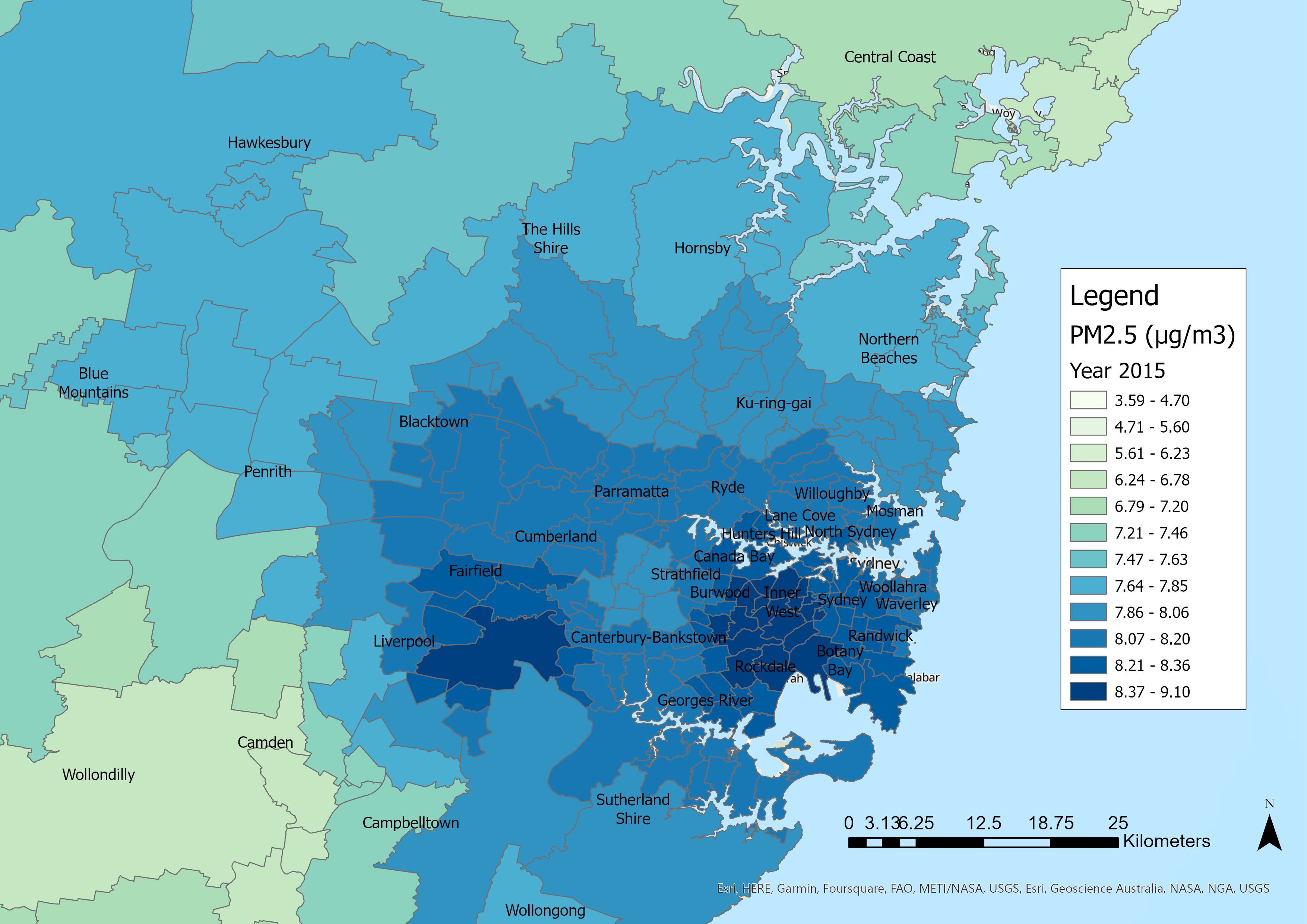


**NO_2_ air pollution by postcode across New South Wales in 2015**

NO_2_ levels were averaged for each postcode across New South Wales using data fusion techniques combining satellite data and ground monitoring station data (see Methods). Annual averages were derived for year of birth, as well as 2015 (year of Middle Childhood Survey). Map of NO_2_ levels for 2015 is shown below.

Supplementary Figure 3. Map of nitrogen dioxide air pollution by postcode across New South Wales in 2015


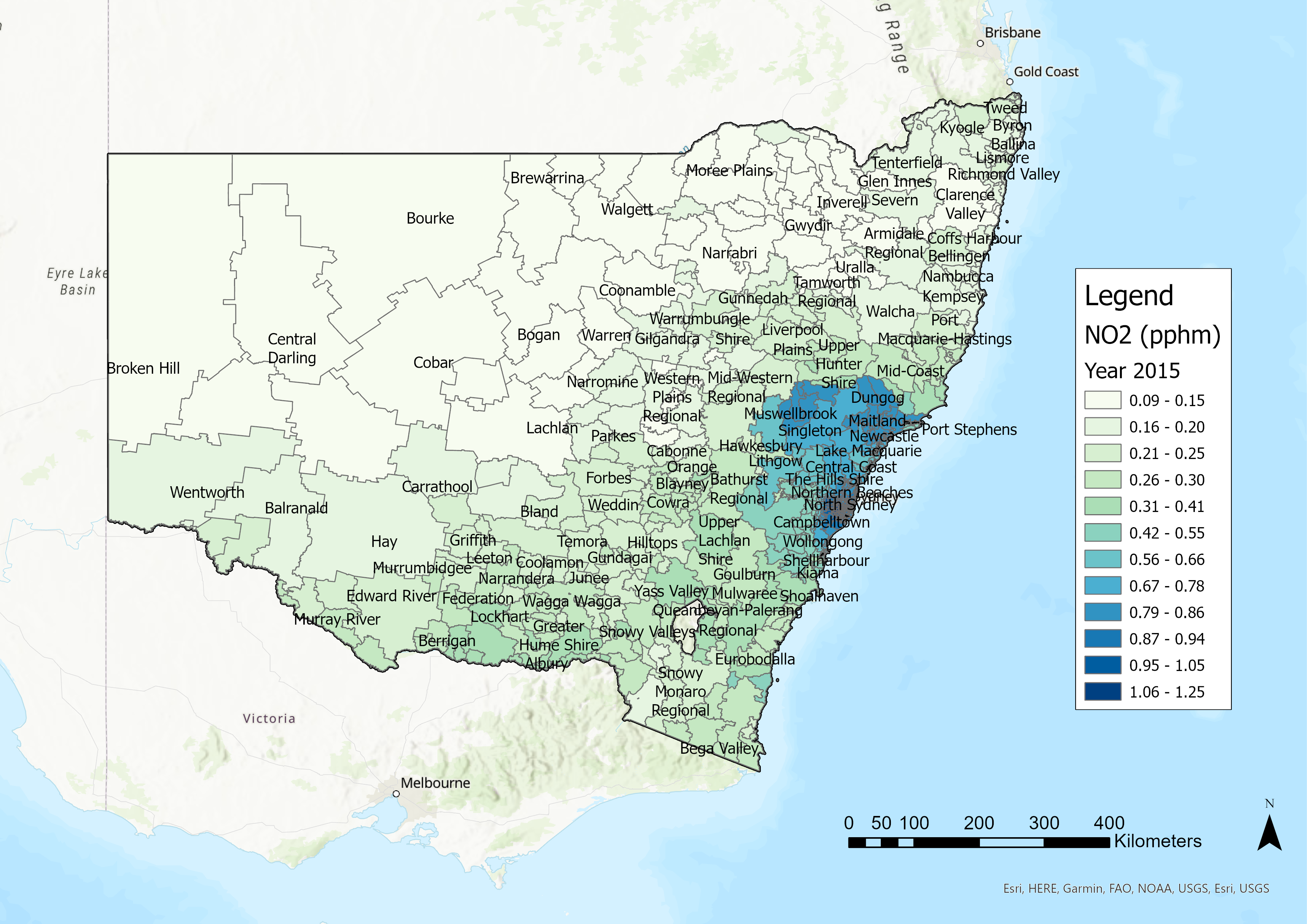


**PM_2.5_ air pollution by postcode across New South Wales in 2015**

PM_2.5_ levels were averaged for each postcode across New South Wales using data fusion techniques combining satellite data and ground monitoring station data (see Methods). Annual averages were derived for year of birth, as well as 2015 (year of Middle Childhood Survey). Map of PM_2.5_ levels for 2015 is shown below.

Supplementary Figure 4. Map of particulate matter (less than 2.5 microns) air pollution by postcode across New South Wales in 2015


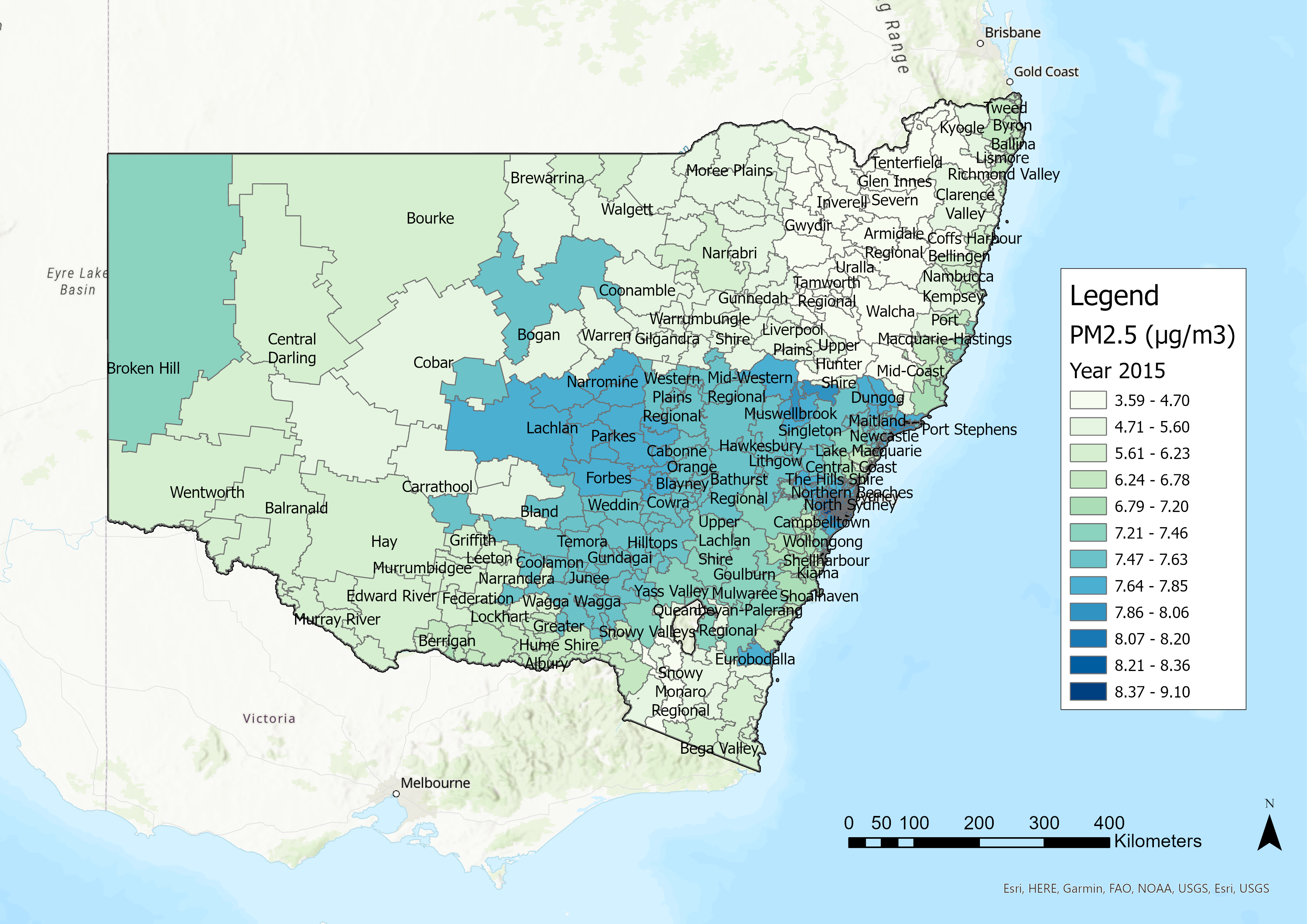


**Sensitivity analysis: Air pollution exposure at birth and middle childhood in association with psychotic-like experiences**

Given the likely correlation between pollution exposures at both birth and middle childhood (since around half the children lived in the same postcode for both timepoints), we conducted further sensitivity analysis that included measures of pollutant exposures at both these time points, in the same model, for the outcome of PLEs as the only outcome significantly associated with air-pollution in the main study. Supplementary Table 1 presents summary statistics for two binomial logistic regression models, one for NO_2_ and one for PM_2.5_, that includes measures of that pollutant exposure at birth and middle childhood, using the same binary outcome variable of PLEs (low or high) and covariates as reported in the primary analysis.

Supplementary Table 1. Birth and middle childhood pollution exposures used simultaneously in a single model with psychotic-like experiences as outcome

|  | | **Odds Ratio (95%CI^[[1]](#footnote-1)^1)** | |
| --- | --- | --- | --- |
| **Air Pollution Variable** | | **NO_2_^[[2]](#footnote-2)^2** | **PM_2.5_^[[3]](#footnote-3)^3** |
| Birth air pollution exposure | | 0.90 (0.80-1.03) | 1.07 (1.02-1.13) |
| Middle Childhood air pollution exposure | | 1.23(1.05-1.44) | 0.97 (0.93-1.02) |
| Socioeconomic status | High | REF | REF |
|  | Low/medium | 1.09 (1.01-1.18) | 1.09 (1.01-1.18) |
| Aboriginal or Torres Strait Islander | No | REF | REF |
|  | Yes | 1.19(1.04-1.36) | 1.18 (1.03-1.36) |
| Parental mental disorder | No | REF | REF |
|  | Yes | 1.09(0.89-1.33) | 1.09(0.89-1.33) |
| Urbanicity | Lowest 25% | REF | REF |
|  | >25% & ≤50% | 1.09(0.97-1.22) | 1.07 (0.95-1.20) |
|  | >50% & ≤74% | 1.05(0.91-1.20) | 1.07 (0.94-1.21) |
|  | Highest 25% | 0.88(0.75-1.04) | 0.94 (0.82-1.06) |

**Sensitivity analysis: Middle childhood exposure to NO_2_ and PM_2.5_ pollution in association with psychotic-like experiences**

Given the correlation between NO_2_ and PM_2.5_ at each time-point (see Table 1 Main Paper), we conducted further sensitivity analysis that included exposures to both NO_2_ and PM_2.5_ in middle childhood, in association with PLEs as the only outcome significantly associated with air-pollution in the main study. Supplementary Table 2 presents summary statistics for a binomial logistic regression model including both NO_2_ and PM_2.5_ exposure in middle childhood, using the same binary outcome variable of PLEs (low or high) and covariates as reported in the primary analysis.

Supplementary Table 2. Adjusted Odd Ratios (and 95% CIs) for exposure to both pollutants in middle childhood in association with psychotic-like experiences

|  | | **Adjusted Odds Ratio (95%CI^[[4]](#footnote-4)^1)** |
| --- | --- | --- |
| Middle Childhood Exposure to NO_2_**^[[5]](#footnote-5)^2** | | 1.06(0.95-1.20)  1.03(0.98-1.08)  REF  1.09(1.01-1.18)  REF  1.19(1.04-1.37)  REF  1.10(0.89-1.33)  REF  1.06(0.95-1.20)  1.03(0.90-1.18)  0.88(0.75-1.04) |
| Middle Childhood Exposure to PM_2.5_**^[[6]](#footnote-6)^3** | |  |
| Socioeconomic status | High |  |
|  | Low/medium |  |
| Aboriginal or Torres Strait Islander | No |  |
|  | Yes |  |
| Parental mental disorder | No |  |
|  | Yes |  |
| Urbanicity | Lowest 25% |  |
|  | >25% & ≤50% |  |
|  | >50% & ≤74% |  |
|  | Highest 25% |  |

**Sensitivity analysis: Use of total scores on the psychotic-like experiences (PLEs) index as an ordinal outcome measure**

This sensitivity analysis explored the use of a total PLE score as the outcome measure instead of a binary (high/low) index that showed small associations with exposure to both NO_2_ and PM_2.5_ in the primary analyses. Using the raw score of PLEs (9 questions based on PLEC-Q, scored as never=0, sometimes=1 and often=2)., a total score was computed (range 0-18; median=5) and considered as an ordinal variable. Supplementary Table 3 presents the results of an ordinal regression analysis of total PLE scores and exposure to NO_2_ and PM_2.5_ at each of the timepoints as the dependent variable. As in the primary analysis, exposure to each pollutant was modelled separately; for each pollutant, there were two models- one for exposure at birth and one for exposure in middle childhood, with the same covariates used as for the main analysis (Aboriginal and Torres Strait Islander status, socioeconomic status, urbanicity and parental mental health history).

Supplementary Table 3. Regression analyses for individual pollutant exposures at birth and at middle childhood using total psychotic-like experience scores as an ordinal outcome

|  | | **Odds Ratio (95%CI^^[[7]](#footnote-7)^1^) for birth exposure** | | | | **Odds Ratio (95%CI^^[[8]](#footnote-8)^1^) for middle childhood exposure** | | | |
| --- | --- | --- | --- | --- | --- | --- | --- | --- | --- |
|  | | **Unadjusted** | | **Adjusted^4^** | | **Unadjusted** | | **Adjusted^4^** | |
| **Variable** | | **NO_2_^^[[9]](#footnote-9)^2^** | **PM_2.5_^^[[10]](#footnote-10)^3^** | **NO_2_** | **PM_2.5_** | **NO_2_** | **PM_2.5_** | **NO_2_** | **PM_2.5_** |
| NO_2_ | | 1.02(0.99-1.04) | - | 1.08(1.03-1.12) | - | 1.03(0.99-1.06) | - | 1.12(1.06-1.18) | - |
| PM_2.5_ | | - | 1.03(1.01-1.05) | - | 1.06(1.03-1.09) | - | 1.05(1.03-1.07) | - | 1.08(1.06-1.11) |
| Socioeconomic status | High | - | - | REF | REF | - | - | REF | REF |
|  | Low/medium | - | - | 1.12(1.06-1.18) | 1.12(1.06-1.18) | - | - | 1.13(1.07-1.19) | 1.13(1.07-1.19) |
| Aboriginal or Torres Strait Islander | No | - | - | REF | REF | - | - | REF | REF |
|  | Yes | - | - | 1.22(1.10-1.34) | 1.22(1.10-1.35) | - | - | 1.22(1.10-1.35) | 1.22(1.11-1.35) |
| Parental mental disorder | No | - | - | REF | REF | - | - | REF | REF |
|  | Yes | - | - | 1.03(0.89-1.19) | 1.03(0.89-1.19) | - | - | 1.03(0.89-1.19) | 1.03(0.89-1.19) |
| Urbanicity | Lowest 25% | - | - | REF | REF | - | - | REF | REF |
|  | >25% & ≤50% | - | - | 1.06(0.97-1.14) | 1.06(0.98-1.14) | - | - | 1.04(0.96-1.13) | 0.99(0.92-1.08) |
|  | >50% & ≤74% | - | - | 0.98(0.89-1.08) | 0.98(0.90-1.06) | - | - | 0.95(0.87-1.05) | 0.93(0.85-1.11) |
|  | Highest 25% | - |  | 0.91(0.82-1.01) | 0.92(0.85-1.00) | - |  | 0.87(0.78-0.97) | 0.88(0.80-0.95) |

1. 1 Confidence interval [↑](#footnote-ref-1)
2. 2 Nitrogen dioxide [↑](#footnote-ref-2)
3. 3 Particulate matter less than 2.5 microns [↑](#footnote-ref-3)
4. 1 Confidence interval [↑](#footnote-ref-4)
5. 2 Nitrogen dioxide [↑](#footnote-ref-5)
6. 3 Particulate matter less than 2.5 microns [↑](#footnote-ref-6)
7. [↑](#footnote-ref-7)
8. 1 Confidence interval [↑](#footnote-ref-8)
9. 2 Nitrogen dioxide [↑](#footnote-ref-9)
10. 3 Particulate matter less than 2.5 microns [↑](#footnote-ref-10)
